# Guanidinoacetic Acid Attenuates Adipogenesis through Regulation of miR-133a in Sheep

**DOI:** 10.3390/ani13193108

**Published:** 2023-10-05

**Authors:** Jia-Min Zhao, Fan-Qin-Yu Li, Xv-Ying Li, Dan-Rong Jiao, Xiang-Dong Liu, Xiao-Yang Lv, Jun-Xing Zhao

**Affiliations:** 1 College of Animal Science, Shanxi Agricultural University, Taigu 030801, Chinaxyl19834541727@163.com (X.-Y.L.); m15834071899@163.com (D.-R.J.); 2 Department of Medical Oncology, Dana-Farber Cancer Institute, Boston, MA 02215, USA; 3 International Joint Research Laboratory in Universities of Jiangsu Province of China for Domestic Animal Germplasm Resources and Genetic Improvement, Yangzhou University, Yangzhou 225009, China

**Keywords:** amino acid, feed additive, lambs, cellular bioenergetics

## Abstract

**Simple Summary:**

Excessive white adipose tissue accumulation in farm animal results in a lower meat percentage of the carcass, which may attenuate economic benefits. Moreover, consumers prefer to purchase lean meat without excessive subcutaneous fat. Thus, developing strategies to enhance skeletal muscle growth and maintain or decrease adiposity in farm animals is desirable. Guanidinoacetic acid (GAA) has been officially registered as an animal feed additive to promote the performance of animals. Currently, little is known about its effects on sheep adipose tissue accumulation. We found that dietary GAA supplementation could attenuate adipose tissue growth in sheep. Moreover, GAA inhibited proliferation and differentiation of sheep stromal vascular fraction (SVF) cells in vitro. Thus, these data could potentiate the application of GAA to sheep meat production.

**Abstract:**

Guanidinoacetic acid (GAA) is an amino acid derivative, previously described in the skeletal muscle of vertebrates, that serves as an important regulator of cellular bioenergetics and has been widely used as a feed additive. Nevertheless, the effect of GAA on adipose tissue growth remains unclear. Here, we hypothesized that dietary GAA negatively affected adipose tissue development in lambs. Lambs were individually fed diets with (0.09%) or without GAA for 70 d ad libitum, and the subcutaneous adipose tissues were sampled for analysis. The results showed that dietary GAA supplementation decreased the girth rib (GR) value (*p* < 0.01) of lamb carcasses. Both real-time PCR and Western blot analysis suggested that dietary GAA inhibited the expression of adipogenic markers, including peroxisome proliferator-activated receptor γ (PPARγ, *p* < 0.05), CCAAT/enhancer-binding protein α (C/EBPα, *p* < 0.01) and sterol-regulatory-element-binding protein 1c (SREBP1C, *p* < 0.01) in subcutaneous adipose tissue. In vitro, GAA inhibited sheep stromal vascular fraction (SVF) cell proliferation, which was associated with downregulation of proliferating cell nuclear antigen (PCNA, *p* < 0.05), cyclin-dependent kinase 4 (CDK 4, *p* < 0.05) and cyclin D1 (*p* < 0.01). GAA suppressed adipogenesis of SVF cells. Furthermore, miRNA sequencing revealed that GAA affected the miRNA expression profile, and real-time PCR analysis confirmed that *miR-133a* expression in both subcutaneous adipose tissue and SVF cell was downregulated by GAA. Meanwhile, miR-133a promoted adipogenic differentiation of SVF cells by targeting *Sirt1*. miR-133a mimics alleviated the inhibitory effect of GAA on SVF cells’ adipogenic differentiation. In summary, GAA attenuated adipogenesis of sheep SVF cells, which might occur through miR-133a-modulated *Sirt1* expression.

## 1. Introduction

Guanidinoacetic acid (GAA) is an amino acid derivative, previously described in the skeletal muscle of vertebrates, and it possesses various biological functions, including energy metabolism regulation, hormonal stimulation, and antioxidant capacity modulation [1]. Moreover, GAA is a metabolic precursor for the biosynthesis of creatine, which has been proven to regulate cellular bioenergetics and has been widely used as a feed additive. In addition, GAA has better stability and higher bioavailability and is more cost-effective than creatine, making it an alternative feed supplement to creatine in farm animal production [2]. Indeed, GAA has been officially registered as an animal feed additive in the US, Europe and China. Previous studies have demonstrated that dietary GAA can effectively promote the growth and development of domestic animals, including pigs [3,4], cattle [5,6], sheep [7,8], and poultry [9,10], and most of these studies were focused on the effects of GAA on skeletal muscle development [11].

Based on anatomical location, white adipose depots in animals can be classified into subcutaneous adipose tissue, visceral adipose tissue, intramuscular adipose tissue and intramuscular adipose tissue. Traditionally, white adipocytes function as the warehouse for excess energy storage and are important endocrine cells [12]. For farm animals, although intramuscular fat contributes positively to carcass value, excessive subcutaneous and visceral adipose tissue accumulation is considered an economic loss for producers. Therefore, understanding the mechanisms of white adipose tissue (WAT) formation in farm animals is necessary. Adipogenesis is a highly orchestrated process that requires the participation of different signaling pathways and transcription factors. MicroRNAs, a class of noncoding RNAs approximately 21–25 nt in length, have been found to be involved in adipocyte differentiation and proliferation [13]. Creatine can negatively regulate adipogenesis. In mouse preadipocytes (C3H10T1/2 and 3T3-L1), creatine inhibits adipogenesis through downregulation of PI3K activity [14], while ablation of adipocyte creatine metabolism causes diet-induced obesity [15]. Although creatine is endogenously produced, its endogenous form may not be sufficient to support the requirements of the animal industry [16], and thus, adequate dietary creatine supplementation might be indispensable.

As mentioned above, GAA has been used as a replacement for creatine in animal feed to regulate skeletal muscle growth and meat quality. Previously, we reported that dietary GAA supplementation in growing lambs improved the skeletal muscle mass and antioxidant capacity of the resulting meat [7]. Whether GAA regulates adipose tissue development remains unknown. We hypothesized that dietary GAA supplementation negatively regulated adipogenesis in lambs, and then investigated the mechanism underlying this effect.

## 2. Materials and Methods

### 2.1. Animal Treatments

The details for the care and use of lambs have been reported in our previously published paper [7]. Briefly, Dorper (♂) × Small-tailed Han sheep (♀) cross male lambs with similar body weights (4 mos of age, 24.8 ± 1.3 kg) were randomly chosen, assigned to two groups (*n* = 12 in each group), individually fed with either a basal diet (control group) or GAA containing diet (GAA group, basal diet contained 0.09% of GAA, dry matter basis) for 70 d ad libitum and had free access to clean water. Prior to the experiment, lambs were fed an experimental diet for 10 d ad libitum for adaptation. After harvest, the tissue depth of the girth rib (GR) was measured and recorded, 110 mm from the midline of the 12th rib, using a Vernier caliper. Meanwhile, subcutaneous white adipose tissues (WATs) of the two groups, at the same anatomical position, were dissected for miRNA sequencing, mRNA and protein extraction.

### 2.2. Stromal Vascular Fraction Cells Isolation and Adipogenic Induction

After harvest, subcutaneous WATs from untreated male lambs were collected for stromal vascular fraction (SVF) cell isolation by following a previously published protocol [17]. Briefly, fresh subcutaneous adipose tissue was washed with cold phosphate buffered saline (PBS, pH 7.4) buffer containing 1% fetal bovine serum (FBS), and visual association and blood vessels were removed using sterile forceps. Next, WAT was minced mechanically with scissors in a 50 mL tube, and cold PBS buffer containing 2000 U/mL collagenase II (Sigma, St. Louis, MO, USA) and 30 U/mL DNase (Sigma, St. Louis, MO, USA). The mixture was digested in a shaking incubator (225 rpm, 37 °C, 30 min). Then, the digested cell suspension was filtered with a 100 μm filter and centrifuged (800× *g*, 10 min, 4 °C) and the cell pellet was resuspended in 500 μL of red blood cell lysate for 1 min. Afterwards, the resuspended cells were transferred to a 50 mL tube and filtered through a 70 μm cell filter. Finally, the cell filtrate was centrifuged (800× *g*, 10 min, 4 °C) and the remaining pellet containing SVF cells was suspended and cultured in a growth medium consisting of 10% FBS and 1% penicillin/streptomycin at 37 °C with 5% CO^2^ and 95% humidity.

For adipogenic induction, SVF cells that reached 100% confluence were cultured in a medium which contained 10% FBS, insulin (2 µg/mL), dexamethasone (1 µM) and IBMX (0.5 mM). The maintenance medium including 10% FBS and insulin (2 µg/mL) was replaced every 2 d.

### 2.3. Oil Red O Staining

The isolated SVF cells were cultured in adipogenic induction medium containing 10% FBS, insulin (2 µg/mL), dexamethasone (1 µM) and IBMX (0.5 mM), with or without GAA (2 mM and 20 mM), for 8 d. Then, the cells were washed with cold PBS twice and fixed with 10% formaldehyde (room temperature, 15 min). After permeation (60% isopropyl alcohol, 1 min), cells were incubated with 0.2% Oil red O solution (Sigma, St. Louis, MO, USA) at room temperature for 1 h. Finally, the cells were rinsed with 60% isopropanol and PBS, and images were taken using a microscope (DMi8, Leica, Wetzlar, Germany).

### 2.4. Wound Healing Assay

The SVF cells were cultured until they reached 100% confluence. Then, the cells were scratched using a sterile pipette tip, and the exfoliated cells were washed using DMEM three times. Subsequently, cells were subjected to either GAA (2 and 20 mM) or a vehicle, and their growth and migration status were monitored using microscopy (Leica, Wetzlar, Germany) at 12, 24, and 36 h after scratching. The circle selection of the scratch area and the percentage of wound closure were calculated by using NIH ImageJ software (Version 1.49).

### 2.5. MTT Assay

Sheep SVF cells seeded on 96-well plates (Logarithmic phase, 20,000 cells/mL) were subjected to either GAA (2 and 20 mM) or a vehicle for different times (0 h, 12 h, 24 h, 36 h and 48 h) and cell viability was measured with a 3-[4,5dimethylthiazol-2-yl]-2,5-diphenyltetrazolium bromide (MTT) assay using a commercial kit (Nanjing Jiancheng Bioengineering Institute, Nanjing, China). Before the test, 10 µL of MTT solution was added to each well and coincubated for 4 h. After that, the supernatant was removed and 100 µL of DMSO was added to each well until the formazan was completely dissolved. Finally, the absorbance (at 570 nm) was measured using a spectrophotometer (Synergy H1 Multi-Mode Reader, Biotek, Winooski, VT, USA).

### 2.6. 5-Ethynyl-2′-deoxyuridine (EdU) Staining

Sheep SVF cells at the logarithmic phase were subjected to either GAA (2 and 20 mM) or a vehicle for 24 h. Then, 50 µM EdU solution was added to the medium and co-incubated for 2 h. After washing with PBS, cells subsequently underwent PFA fixation (4%, room temperature, 30 min), permeabilization (0.5% Triton X-100, room temperature, 5 min), and coincubation with 1× Apollo reaction cocktail (1 h, room temperature). Nuclei were stained using DAPI dye solution, and EdU-positive cells were detected using a microscope (DMi8, Leica, Wetzlar, Germany).

### 2.7. RNA Preparation, Library Construction, and Sequencing

Total RNA from WAT was extracted using TRIzol reagent (Sigma, St. Louis, MO, USA), the concentration and purity were measured by a NanoDrop 2000 instrument (Nanodrop Instruments, Wilmington, DE, USA) and the integrity was evaluated by agarose gel electrophoresis and an Agilent 2100 Bioanalyzer (Agilent Technologies, Inc., Santa Clara, CA, USA). Only high-quality RNA samples (OD260/280 = 1.8~2.0, OD260/230 ≥ 2.0, RIN > 6.5, 28S:18S ≥ 1.0, >1 μg) were used to construct the sequencing library. RNA of 18–30 nt was isolated and purified from the total RNA using denaturing polyacrylamide gel. miRNA sequencing (miRNA-seq) libraries were constructed using a QIAseq miRNA Library Kit (Qiagen, Hilden, Germany) following manufacturer’s recommendations, and the generated library fragments were sequenced on an Illumina HiSeq-4000 platform with a mean sequencing depth of 10 million reads (50 bp single-end reads) by Gene Denovo Biotechnology Co., Ltd. (Guangzhou, China).

### 2.8. Bioinformatics Analysis

Bioinformatics analysis was performed by Gene Denovo Biotechnology Co., Ltd. (Guangzhou, China). Briefly, the clean reads were compared to the Genbank (http://www.ncbi.nlm.nih.gov, accessed on 15 December 2022) and Rfam databases (http://rfam.xfam.org, accessed on 22 December 2022) to filter out the non-coding RNAs. Then, the remaining clean reads were aligned to the sheep miRNA precursors presented in miRBase (http://www.mirbase.org/, accessed on 13 January 2023) using miRDeep (v2.0.0.8). Differentially expressed miRNAs were identified by edgeR (https://bioconductor.org/packages/release/bioc/html/edgeR.html, accessed on 31 January 2023; (|log2(FoldChange)| ≥ 1 and FDR < 0.05).

### 2.9. Western Blotting

Western blotting was conducted by following a published method in our laboratory [18]. Briefly, cells were lysed using M5 RIPA Lysis Buffer (Mei5bio, Beijing, China), adipose tissue was lysed using the M5 HiPer Tissue Protein Extraction Kit (Mei5bio, Beijing, China) and lysate supernatant was separated by centrifugation (12,000× *g*, 10 min at 4 °C) and boiled for 10 min on a thermal cycler at 95 °C. Proteins were used for electrophoresis using a 10% SDS-PAGE gel (4 °C, 120 V for 2 h), and separated proteins were transferred to a nitrocellulose (NC) membrane (4 °C, 100 V for 1.5 h). Then, the NC membrane was blocked with 5% skimmed milk solution for 1 h and washed with TBST solution. After that, the membrane was incubated with primary antibody (at 4 °C overnight) and secondary antibody (1 h, room temperature). Target proteins were scanned using an Odyssey Infrared Imaging System (LI-COR Biosciences, Lincoln, NE, USA), and β-actin content was used for normalization.

Antibody information is shown below: proliferating cell nuclear antigen (#2586S, PCNA) was purchased from Cell Signaling (Danvers, MA, USA). Cyclin D1 (bs-20596R), cyclin-dependent kinase 4 (bs-0633R, CDK4), peroxisome proliferator-activated receptor γ (bs-4888R, PPARγ), CCAAT/enhancer-binding protein α (bs-1630R, C/EBPα), sterol-regulatory-element-binding protein 1c (bs-1402R, SREBP1c), acetyl-CoA carboxylase (bs-2745R, ACC), NAD-dependent deacetylase sirtuin-1 (bs-2257R, SIRT1), fatty acid binding protein 4 (bs-4059R, FABP4) and β-actin (bs-0061R) were obtained from Biosynthesis Biotechnology Co., Ltd. (Beijing, China). Secondary antibodies (Goat anti-rabbit) were from LI-COR Biosciences (Lincoln, NE, USA).

### 2.10. Quantitative Real-Time PCR Analysis

Total RNA was extracted using TRIzol reagent (Sigma, St. Louis, MO, USA), and the quality (with OD 260/280 ≥ 1.8 and OD 260/230 ≥ 2.0 were used) and concentration of RNA were assessed using a NanoDrop 2000 instrument (Nanodrop Instruments, DE, USA). PrimeScript™ RT Master Mix kit (Takara Co., Ltd., Dalian, China) and miRNA 1st strand cDNA synthesis Kit (Vazyme Biotech Co., Ltd., Nanjing, China) were used for reverse transcription into mRNA and miRNA, respectively. Quantitative real-time PCR analysis (qRT-PCR) was performed using TB Green™ Premix Ex Taq™ (Takara Co., Ltd., Dalian, China) and miRNA Universal SYBR qPCR Master Mix (Vazyme Biotech Co., Ltd., Nanjing, China), and the 2^−ΔΔCt^ method was applied to assess the relative expression of all RNAs; *U6* and 18 s rRNA served as housekeeping genes for miRNA and mRNA quantification, respectively (Table 1).

### 2.11. Cell Transfection

Transfection was performed when cells reached 60% confluence. The transfections were divided into four groups: miR-133a mimics, mimic negative control, miR-133a inhibitor and inhibitor negative control. For each transfection, oligonucleotides and Lipofectamine 3000 (Invitrogen Life Technologies, Carlsbad, CA, USA) were mixed with 100 µL Opti-MEM medium (Gibco, Carlsbad, CA, USA) and incubated for 5 min, respectively. Then, the two mixtures were mixed and incubated for 10 min and, after that, the mixture was added to the culture medium. After culturing for 6 h, the medium was replaced with growth medium, and the cells were cultured for further use. The miR133a mimics (TTTGGTCCCCTTCAACCAGCTG), negative control (NC; TTCTCCGAACGTGTCACGTTT), miR133a inhibitor (CAGCTGGTTGAAGGGGACCAAA) and miR133a inhibitor NC (CAGTACTTTTGTGTAGTACAA) were purchased from Jima Pharmaceutical Technology (Shanghai, China).

### 2.12. Dual Luciferase Assay

A fragment of the sheep SIRT1 3′-untranslated region (UTR) sequence (4320 to 5020 bp) was cloned into the psiCHECK-2 dual-luciferase reporter plasmid by Wuhan GeneCreate Biological Engineering Co., Ltd. to generate SIRT1-3′UTR wild type (WT) (Wuhan, China). Meanwhile, the SIRT1-3′UTR mutant was obtained from the same company. After that, the binding potential of miR-133a and its target genes were verified by a dual-luciferase reporter kit (Promega, Madison, WI, USA). Briefly, human 293T cells seeded in 96-well plates were simultaneously transfected with miR-133a mimics + 3′UTR-WT, mimics NC + 3′UTR-WT, mimics + 3′UTR-MUT and mimics NC + 3′UTR-MUT using Lipofectamine 3000 reagent. Forty-eight hours after transfection, cells were sampled and luciferase activities were detected by using a Synergy H1 microplate reader (Biotek, Winooski, VT, USA).

### 2.13. Triglyceride Measurement

The triglyceride (TG) measurement was performed using a TG test kit (A110-1-1, Nanjing Jiancheng Bioengineering Institute, Nanjing, China) in accordance with the manufacturer’s instructions. Briefly, SVF cells seeded in six-well plates were induced to adipogenesis with or without GAA. Then, the cells were collected and lysed in 1% Triton X-100, and the absorbance value was obtained with a Synergy H1 microplate reader (BioTek, Winooski, VT, USA).

### 2.14. Statistical Analysis

All experimental data were displayed as the mean ± SEM, and in vivo data were analyzed using a linear mixed model with SPSS (IBM SPSS Statistics for Windows, Version 22.0). In the statistical model, the treatment was the fixed variable, and animal ID and pen were considered as random variables. In vitro data were analyzed using Student’s *t*-test with GraphPad Prism 8 Software (San Diego, CA, USA). Unpaired *p* < 0.05 was identified as a statistically significant difference.

## 3. Results

### 3.1. Dietary Effects of GAA on Body Fatness 

**Figure 1:**
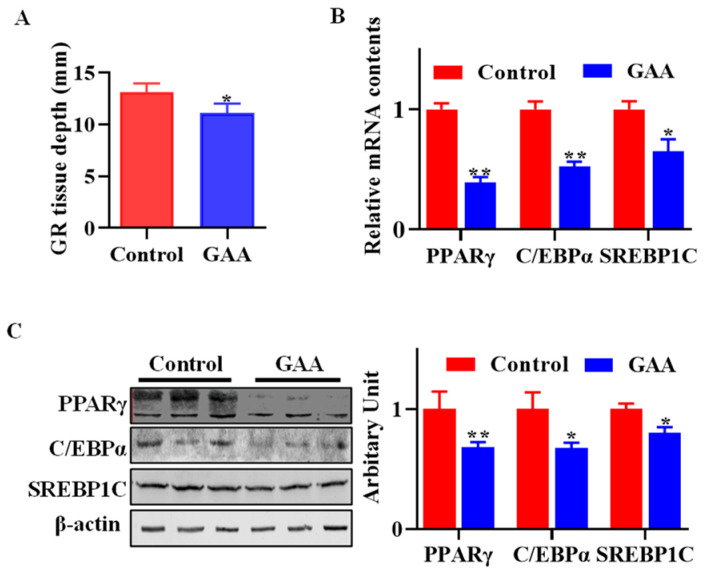
GAA reduced girth rib (GR) value and attenuated adipogenic transcriptional factors expression in subcutaneous white adipose tissue (WATs)**.** Lambs were fed with a basal diet (control group) and GAA containing diet (GAA group, basal diet contained 0.09% of GAA, dry matter basis). After harvest, the tissue depth of the GR was measured, and WATs at the same anatomical position were dissected for mRNA and protein extraction. (**A**) GR value of carcass. (**B**) Relative mRNA expression of *PPARγ*, *C/EBPα* and *SREBP1C* in subcutaneous WATs. (**C**) PPARγ, C/EBPα and SREBP1C protein abundances in subcutaneous WATs. (*n* = 12, mean ± SEM; * *p* < 0.05, ** *p* < 0.01).

The GR value has been widely used as an indicator of carcass fatness [19]. GR tissue depth was lower in lambs fed GAA (*p* < 0.05, Figure 1A). Moreover, dietary GAA effectively downregulated the expression of key adipogenic genes, including *PPARγ* (*p* < 0.01), *C/EBPα* (*p* < 0.01) and *SREBP1C* (*p* < 0.05) (Figure 1B). Consistently with this, the protein abundances of PPARγ (*p* < 0.01), C/EBPα (*p* < 0.05) and SREBP1C (*p* < 0.05) (Figure 1C) were decreased when lambs were fed GAA.

### 3.2. GAA Inhibited SVF Cell Proliferation 

**Figure 2:**
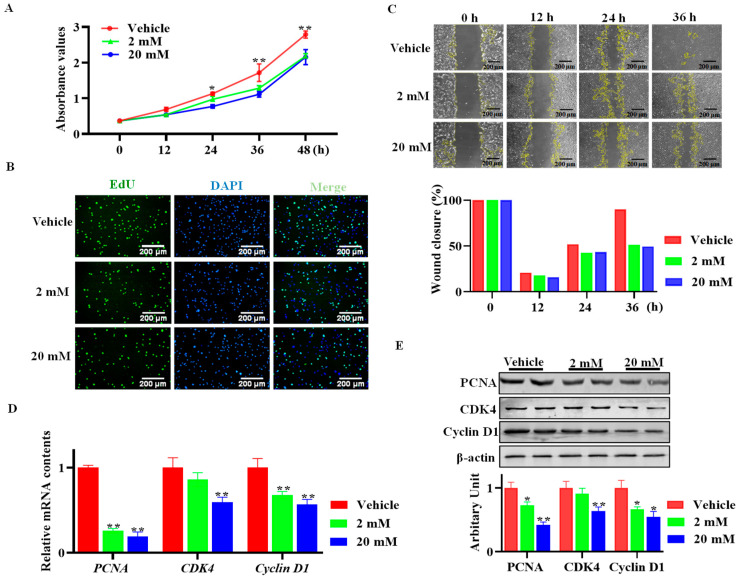
GAA inhibited stromal vascular fraction (SVF) cells’ proliferation. Isolated SVF cells were treated with either GAA (2 and 20 mM) or a vehicle. (**A**) MTT assay. (**B**) EdU staining. (**C**) Wound healing assay. (**D**) Relative mRNA expression of *PCNA*, *CDK4* and *Cyclin D1*. (**E**) PCNA, CDK4 and Cyclin D1 protein abundances. (Scar bar = 200 μm; mean ± SEM; *n* = 6; * *p* < 0.05, ** *p* < 0.01).

As shown in Figure 2A, the sheep SVF cells subjected to GAA exhibited decreased proliferation ability, and 2 mM GAA was sufficient for inhibition (Figure 2A). Meanwhile, the EdU staining results suggested that both 2 mM and 20 mM GAA decreased the number of EdU-positive cells (Figure 2B). Furthermore, the wound healing assay clearly confirmed the inhibitory effect of GAA on sheep SVF cells (Figure 2C). Compared with the control group, sheep SVF cells that received both 2 mM and 20 mM GAA showed decreased *PCNA* (*p* < 0.01) and *Cyclin D1* (*p* < 0.01) mRNA contents, while 20 mM GAA downregulated *cdk4* (*p* < 0.01) mRNA expression (Figure 2D). Consistently with this, GAA reduced the protein abundances of PCNA, CDK4 and Cyclin D1 (Figure 2E).

### 3.3. GAA Attenuated SVF Cell Adipogenic Differentiation 

**Figure 3:**
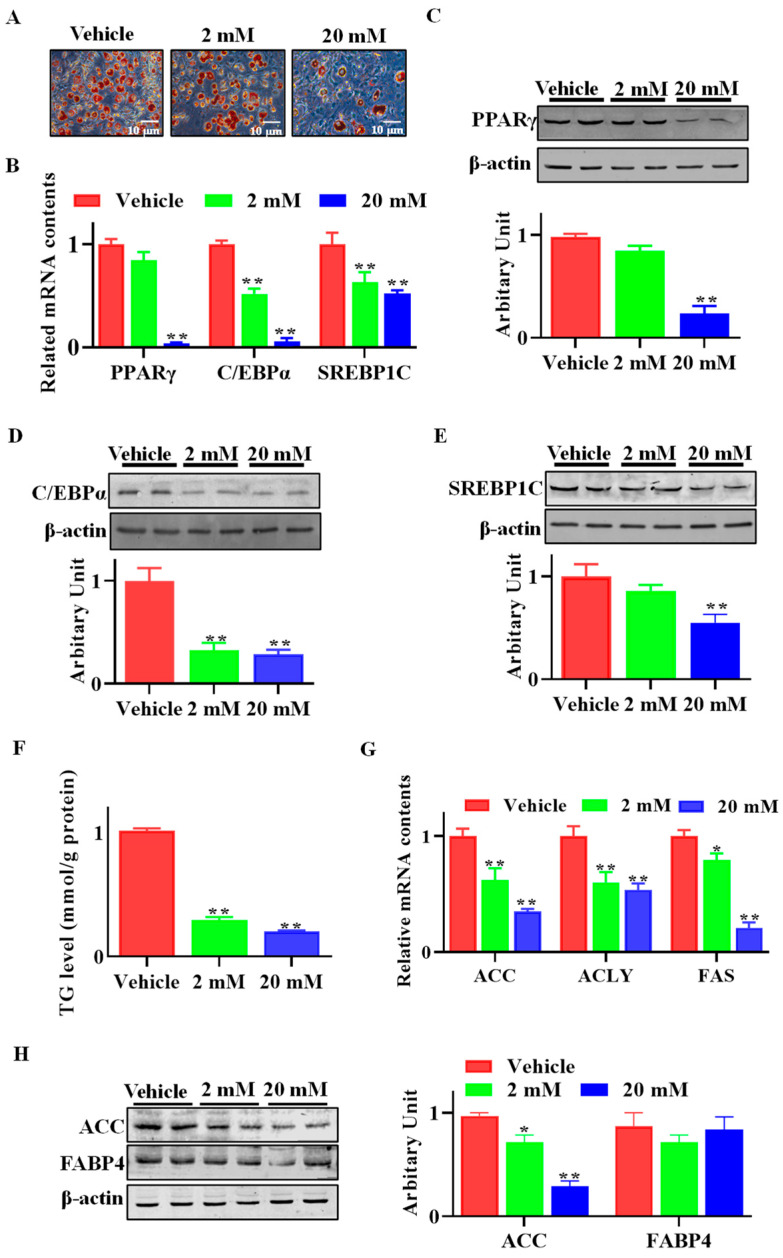
GAA attenuated stromal vascular fraction (SVF) cells’ adipogenic differentiation. Sheep SVF cells were induced to white adipogenesis for 8 d with or without GAA (2 and 20 mM). (**A**) Adipocytes were visualized by Oil red O staining. (**B**) Relative mRNA expression of *PPARγ*, *C/EBPα* and *SREBP1C*. (**C**–**E**) Protein abundances of PPARγ, C/EBPα and SREBP1c. (**F**) Triglyceride contents in mature adipocytes. (**G**) Relative mRNA expression of *ACC*, *ACLY* and *FAS*. (**H**) ACC and FABP4 protein abundances. (Scar bar = 10 μm; mean ± SEM; *n* = 6; * *p* < 0.05, ** *p* < 0.01).

As shown in Figure 3A, both 2 mM and 20 mM of GAA dramatically inhibited lipid accumulation in differentiated SVFs compared with control cells. The mRNA levels of both *C/EBPα* and *SREBP1C* were downregulated when SVF cells were treated with either 2 mM or 20 mM GAA, and 20 mM GAA reduced *PPARγ* mRNA abundance (Figure 3B). Accordingly, the protein abundances of master transcription factors, including PPARγ, C/EBPα and SREBP1C were decreased when SVF cells were subjected to GAA (Figure 3C–E). Compared with the control group, cells treated with GAA showed lower triglyceride (TG) contents (*p* < 0.01, Figure 3F). Furthermore, compared with the control cells, the mRNA expression of *ACC* (*p* < 0.01), *ACLY* (*p* < 0.01) and *FAS* (*p* < 0.01) declined when cells were subjected to GAA (Figure 3G). Although FABP4 protein content did not vary between control and GAA-treated cells, decreased ACC content was observed in the GAA-treated group (Figure 3H).

### 3.4. Effects of GAA on miR-133a and Sirt1 Expression

**Figure 4:**
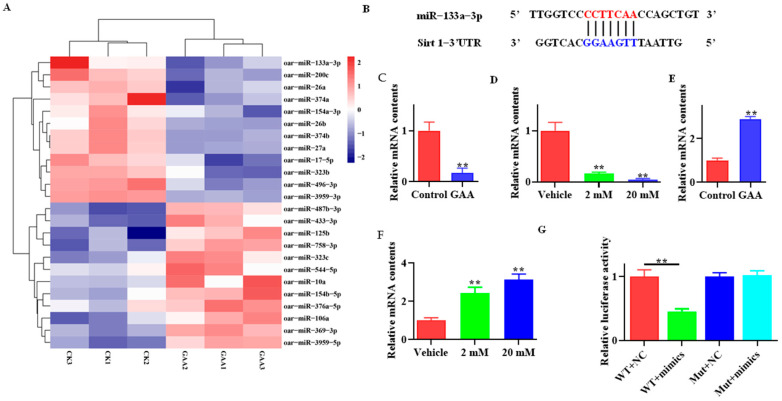
Effects of GAA on miR-133a and SIRT1 expression. (**A**) Heatmap of miRNA expression in subcutaneous WAT of lambs fed or not fed GAA. y-axes indicate the miRNA expression in six samples; CK denotes control lambs. The higher and lower expression of miRNA are shown with red and blue colors, respectively. (**B**) Predicted binding site of the miR-133a on 3′UTR of *Sirt1*. (**C**,**D**) mRNA expression of *miR-133a* in subcutaneous WAT (**C**) and SVF cells (**D**). (**E**) Relative mRNA expression of *Sirt1* in subcutaneous WAT (**E**) and SVF cells (**F**). (**G**) Human 293 T cells were transfected with a dual luciferase reporter (wild type and mutant), together with miR-133a-3p mimics or native control. Data show relative luciferase activities. (mean ± SEM; ** *p* < 0.01).

miRNA sequencing revealed a variety of differentially expressed miRNAs, and the top 12 upregulated and 12 downregulated miRNAs are shown in Figure 4A. Interestingly, a potential binding site of miR-133a was predicted in the 3′UTR of *Sirt1* (Figure 4B). Therefore, the expressions of miR-133a were validated in both subcutaneous adipose tissue (Figure 4C, *p* < 0.01) and SVFs cells (Figure 4D, *p* < 0.01) using qRT-PCR, and the results suggested that GAA downregulated miR-133a expression, both in vivo and in vitro. As expected, *SIRT1* mRNA levels were elevated in both subcutaneous adipose tissue (Figure 4E, *p* < 0.01) and SVF cells (Figure 4F, *p* < 0.01). Furthermore, cells transfected with miR-133a mimics exhibited lower luciferase activity than control cells (Figure 4G, *p* < 0.01).

### 3.5. Effect of miR-133a on Adipogenic Differentiation of Sheep SVF Cells

**Figure 5:**
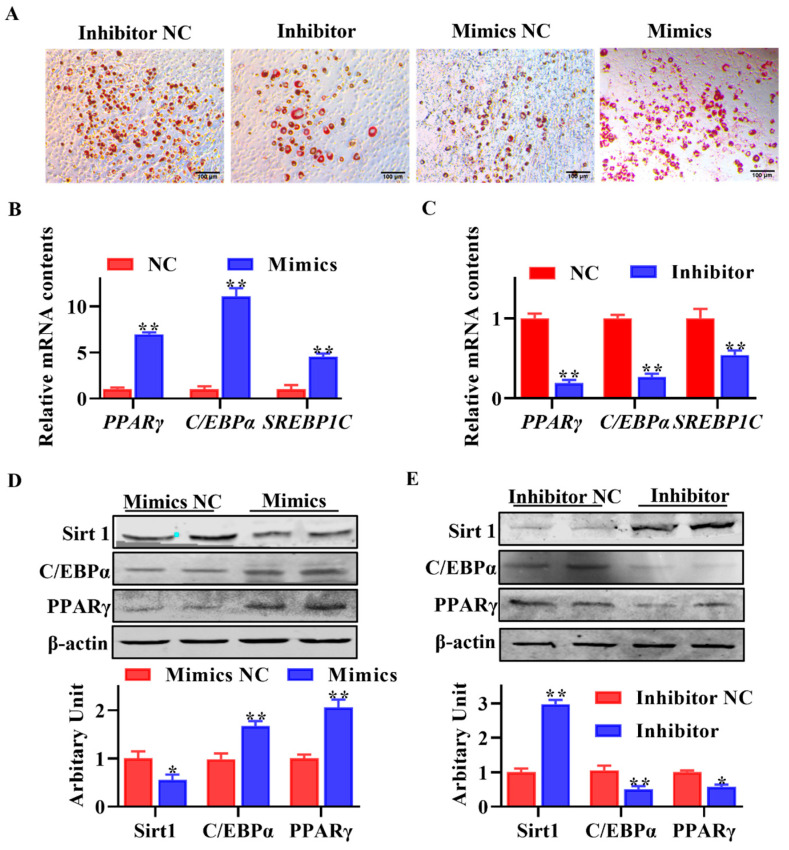
miR-133a inhibited adipogenic differentiation of sheep stromal vascular fraction (SVF) cells. (**A**) Sheep SVF cells transfected with miR-133a inhibitor, inhibitor NC, miR-133a mimics and mimics NC were induced to adipogenesis for 8 d, and adipocytes were visualized by Oil red O staining. (**B**,**C**) Relative mRNA expression of *PPARγ*, *C/EBPα* and *SREBP1C* between groups. (**D**,**E**) Protein abundances of SIRT1, PPARγ and C/EBPα between groups. (Scar bar = 100 μm; mean ± SEM; *n* = 6; * *p* < 0.05, ** *p* < 0.01).

As Figure 5A shows, more lipid droplets were observed when SVF cells were transfected with miR-133a mimics, while lipid accumulation was reduced in miR-133a inhibitor transfected cells. Moreover, qPCR results indicated that miR-133a overexpression promoted *PPARγ*, *C/EBPα* and *SREBP1C* gene transcription (Figure 5B, *p* < 0.05), and these gene expression levels were decreased when SVF cells were transfected with miR-133a inhibitor (Figure 5C, *p* < 0.05). SVF cells transfected with miR-133a mimics exhibited lower SIRT1 (*p* < 0.05), and greater PPARγ (*p* < 0.01) and C/EBPα (*p* < 0.01) abundances (Figure 5D). Conversely, greater SIRT1 (*p* < 0.01), and lower PPARγ (*p* < 0.01) and C/EBPα (*p* < 0.05) protein contents were observed when cells were transfected with miR-133a inhibitor (Figure 5E). Collectively, these results indicated that miR-133a positively regulated adipogenesis in sheep SVF cells.

### 3.6. GAA Attenuated Adipogenesis via Regulation of miR-133a

**Figure 6:**
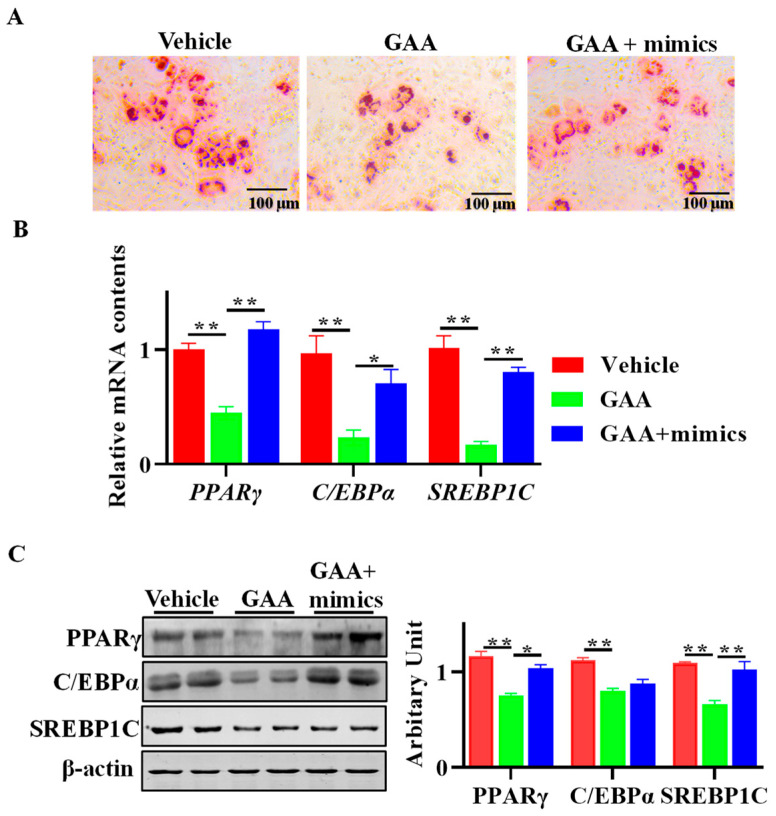
GAA attenuated adipogenesis via regulation of miR-133a expression. (**A**) Sheep SVF cells treated with a vehicle, GAA and GAA + miR-133 mimics were induced to white adipogenesis for 8 d, and mature adipocyte was stained by Oil Red O. (**B**,**C**) Relative mRNA (**B**) and protein (**C**) contents of PPARγ, C/EBPα and SREBP1C. (Scar bar = 50 μm; *n* = 6; * *p* < 0.05, ** *p* < 0.01).

As Figure 6A shows, Oil red O staining indicated that the inhibitory effect of GAA on the adipogenic differentiation of sheep SVF cells was reversed when miR-133a was overexpressed. Moreover, the inhibitory effect of GAA on PPARγ, C/EBPα and SREBP1C expression at both the mRNA (Figure 6B) and protein levels (Figure 6C) was attenuated when the GAA-treated cells were transfected with miR-133a mimics.

## 4. Discussion

In farm animals, the skeletal muscle growth rate is considered a major determinant of performance, since it positively affects the efficiency of meat production and economic benefits [20]. Consequently, studies related to farm animal skeletal muscle growth and development have been extensively performed, and indeed, one of the major aims for animal breeders and animal nutritionists is to promote skeletal muscle growth [21]. Adipose tissues play a key role in whole body energy metabolism and function as endocrine organs that secrete a variety of adipokines for physiological and metabolic regulation [22]. From a carcass perspective, excessive adipose tissue accumulation in farm animal results in a lower meat percentage of the carcass, which further attenuates economic benefits. Thus, developing strategies to enhance skeletal muscle growth and maintain or decrease adiposity in farm animals is desirable.

As mentioned above, GAA has been officially registered as an animal feed additive that has been proven to promote the performance of animals (including pigs, sheep, cattle and poultry) [11]. Our previous results demonstrated that dietary GAA supplementation affected sheep skeletal muscle mass and meat quality [7]. Herein, we continue to determine the effect of GAA on sheep adipose tissue accumulation. A lower GR tissue depth was observed in lambs fed the GAA diet. Given that GR tissue depth is an important indicator of sheep carcass fatness [19], the results suggested that dietary GAA inclusion could attenuate adipose tissue growth in lambs, in keeping with a recent report indicating that dietary GAA supplementation decreases backfat thickness and serum TG in rapid-growing lambs [8]. For sheep meat, the retail value is negatively related to fatness, because an overfat carcass decreases the proportion of saleable lean meat and increases its trimming cost [23]. Moreover, consumers prefer to purchase lean meat, without excessive subcutaneous adipose tissue. On the other hand, a certain level of fatness is necessary for lamb meat quality, and a minimum of 6 mm in the carcass is recommended by Meat Standards Australia [24]. In the current experiment, the average GR tissue depth was 11.2 mm in GAA-fed lambs, suggesting that the application of GAA in the lamb industry could reduce subcutaneous fat deposition without affecting meat quality.

Accumulation of adipose tissue relies on adipogenesis, and as expected, the abundances of C/EBPα, PPARγ and SREBP1c declined in subcutaneous adipose tissue of GAA fed lambs, which further confirms the inhibitory role of GAA on adipose tissue growth. Adipogenesis requires a crosstalk between cell proliferation and cell differentiation [25]. SVFs isolated from adipose tissue are mixtures containing multiple progenitor cells, including preadipocytes, pericytes, adipose-derived stem/stromal cells and mesenchymal stem cells, and have been widely used as in vitro models to recapitulate adipogenesis. The wound healing scratch assay is a standard in vitro technique to evaluate cell migration and proliferation [26], while EdU is commonly used as a cell proliferation probe [27]. In the present study, we found that 2 mM GAA was sufficient to inhibit SVF cell proliferation in vitro, which was accompanied by the downregulation of PCNA, Cyclin D1 and CDK4. Given that these three proteins are key regulators of cell proliferation, the antiproliferative effect of GAA might be due to its regulation of Cyclin D1, CDK4 and PCNA expression. Similarly, GAA inhibits C2C12 cell proliferation, which is accompanied by downregulated expression of CyclinD1 and CDK4 [28]. Mitotic clonal expansion is a prerequisite for preadipocytes terminally differentiating into adipocytes [29], and thus, the decreased proliferative ability in GAA-treated SVF cells suggested that adipogenesis was compromised. Previously, GAA has been proven to exert a negative regulatory effect on adipogenesis in both mice and human mesenchymal stem cell lines by inhibiting PI3K-Akt-PPARγ signaling [14]. To further confirm the effect of GAA on adipogenesis, sheep SVF cells were treated with GAA or not treated, and as expected, attenuated adipogenesis was observed when SVF cells were subjected to GAA.

The next question is how GAA regulates sheep adipogenesis. miRNAs are important for animal growth and development, and several miRNAs that are involved in adipocyte differentiation and fat metabolism have been identified [30]. Likewise, GAA may regulate myogenic differentiation and muscle growth in mice through interaction with miRNAs [28]. Given that various exogenous nutrients affect physiological functions through the regulation of the miRNA expression, we wanted to know whether miRNAs were involved in GAA-regulated adipogenesis. In the present study, 39 differentially expressed miRNAs were identified in lamb subcutaneous adipose tissue. Previous studies have demonstrated that SIRT1 inhibits adipogenesis in both mouse C3H10T1/2 pluripotent cells and porcine preadipocytes [31,32]. In addition, a recent report showed that SIRT1 activity positively correlates with GAA content [33]. miR-133a is highly conserved among species and was initially found to regulate skeletal muscle [34] and heart development [35]. Recently, miR-133a has been found to be expressed in subcutaneous white adipose tissue and brown adipose tissue, and to regulate brown adipose tissue formation [36]. Interestingly, a miR-133a targeting site was predicted in the 3′UTR of *Sirt1*, therefore, miR-133a was chosen for further study. Through validation, we confirmed that GAA downregulated miR-133a and upregulated *Sirt1* expression in both subcutaneous adipose tissue and SVF cells. Moreover, a dual luciferase assay further suggested the relationship between *Sirt1* and miR-133a. Overexpression of miR-133a in sheep SVF cells significantly increased and, conversely, inhibition of miR-133a decreased SIRT1 contents and adipogenesis. Considering that SIRT1 suppresses adipogenesis through inhibition of PPARγ activity [37] and activation of Wnt/β-catenin signaling in white adipose tissue [38], these data suggested that miR-133a positively regulated adipogenesis through modulation of *Sirt1* in sheep SVF cells. Similarly, a recent report from Li et al. suggests that miR-133a serves as a positive regulator of adipogenesis in goat subcutaneous preadipocytes [39]. To further elucidate whether GAA suppresses adipogenesis through regulation of miR-133a, sheep SVF cells treated with GAA were transfected with miR-133a mimics. The inhibitory effect of GAA on sheep SVF cell adipogenesis was compromised, suggesting that the inhibitory effect of GAA might occur, if not entirely, through regulation of miR-133a.

As mentioned before, GAA has been used as a dietary supplement to enhance growth performance of livestock, promote skeletal muscle development and improve the health of animals [11]. To the best of our knowledge, this is the first time that GAA has been proved to affect adipose tissue growth in lambs, and this may provide a new strategy to manipulate fat deposition in sheep. Whether the response to GAA supplementation varies among sheep breeds and among different production systems needs to be further explored.

## 5. Conclusions

In summary, dietary GAA supplementation reduced GR tissue depth and inhibited adipogenesis of subcutaneous adipose tissue. Moreover, GAA inclusion in the diet affected miRNA profiles in subcutaneous adipose tissue. In vitro, GAA exerted an antiproliferative effect and suppressed adipogenesis of sheep SVF cells, which might occur through miR-133a-modulated *Sirt1* expression.

## Figures and Tables

**Table 1:** The primer sequences.

Gene		Sequence (5′-3′)
*PCNA*	Forward	GAGGCGTCTCAGGCGTTC
	Reverse	TTAAGCGCCTCCAGCACTTT
*Cdk4*	Forward	GCCCCGAGATGTCTCTCTAC
	Reverse	AGAGATTCGCTTGTGTGGGT
*CyclinD1*	Forward	GTCCTGGTGTTTTCCTTTGCTC
	Reverse	CTCTTCCCTCTCTTCAGCTCAG
*PPARγ*	Forward	CCGGCGCAGTTGTTCAG
	Reverse	CGGCATCTCTGTGTCAACCA
*C/EBPα*	Forward	AGACGTCCATCGACATCAGC
	Reverse	CCCGGGTAGTCAAAGTCGTT
*SREBP1C*	Forward	GCCTTCTATTACATCCACAACCTTG
	Reverse	AGATTATCCAGCATCCGCATGAG
*ACC*	Forward	CTCGCCCTCAACGTAGGAAG
	Reverse	GAACACATCGAGGGGAAGCA
*ACLY*	Forward	TAACACCATCATCTGTGCTCGG
	Reverse	GTCGAAGGCCTTGCTGAACA
*FAS*	Forward	CTGGTGACGGCCAACTGTAT
	Reverse	CACTGGCCCTGGGTTATGTT
*Sirt 1*	Forward	ACACCTGTCACTGTGGTAGAG
	Reverse	GAACCGTAACCGGGGTCTG
*miRNA-133a*	Forward	CGTTTGGTCCCCTTCAACC
	Reverse	AGTGCAGGGTCCGAGGTATT
*U6*	Forward	CTCGCTTCGGCAGCACA
	Forward	AACGCTTCACGAATTTGCGT
*18S rRNA*	Forward	CGGCTACCACATCCAAGGAA
	Reverse	GCTGGAATTACCGCGGCT

## Data Availability

All data are available in the article.
